# Glomerular Capillary Microaneurysms in Membranoproliferative Glomerulonephritis: A Clinicopathological Study Highlighting the Involvement of IgG3

**DOI:** 10.1111/pin.70144

**Published:** 2026-06-25

**Authors:** Akiko Mii, Tomohiro Kaneko, Momoko Arai, Takehisa Yamada, Mayuko Takeuchi, Tetsuya Kashiwagi, Naomi Kuwahara, Arimi Ishikawa, Ryuji Ohashi, Yukinao Sakai, Masato Iwabu, Akira Shimizu

**Affiliations:** ^1^ Department of Endocrinology, Metabolism and Nephrology Nippon Medical School Tokyo Japan; ^2^ Department of Analytic Human Pathology Nippon Medical School Tokyo Japan; ^3^ Department of Integrated Diagnostic Pathology Nippon Medical School Bunkyō Japan

**Keywords:** endothelial injury, glomerular capillary microaneurysms, IgG3, membranoproliferative glomerulonephritis

## Abstract

Glomerular capillary microaneurysm (GCM) is a pathological lesion defined by aneurysmal dilatation of glomerular capillaries resulting from mesangiolysis with severe endothelial damage and is commonly observed in diabetic nephropathy and thrombotic microangiopathy. However, its clinicopathological significance in membranoproliferative glomerulonephritis (MPGN)‐pattern glomerular diseases remains unclear. We retrospectively reviewed 50 renal biopsy cases diagnosed as MPGN or MPGN‐like glomerulopathy and identified 12 GCM‐positive cases for clinicopathological analysis, including Pathologische Anatomie Leiden Endothelium (PAL‐E) staining and CD34 immunohistochemistry to assess endothelial injury. Among GCM‐positive cases, proliferative glomerulonephritis with monoclonal immunoglobulin G deposits (PGNMID) was significantly enriched; however, exclusive glomerular IgG3 positivity was consistently observed across other diagnostic categories, irrespective of light‐chain restriction. Electron microscopy revealed continuous, irregularly shaped, fine granular subendothelial electron‐dense materials with endothelial denudation. PAL‐E staining was positive in all evaluable cases, with ultrastructurally identified diaphragmed endothelial fenestrae. Furthermore, CD34 staining demonstrated loss of continuous lining in GCM lesions, suggesting severe endothelial injury. Clinically, all patients presented with nephrotic‐range proteinuria, and more than half progressed to end‐stage kidney disease or died within 3 years. These findings indicate that GCM‐positive MPGN‐pattern glomerular diseases represent an aggressive subset associated with IgG3‐dominant deposition and severe endothelial injury, with important diagnostic implications.

## Graphical abstract

Glomerular capillary microaneurysms (GCMs) were most frequently observed in proliferative glomerulonephritis with monoclonal immunoglobulin G deposits (PGNMID) among MPGN‐pattern glomerular diseases, but were not restricted to this entity. These lesions were characterized by exclusive IgG3‐dominant glomerular deposition, irrespective of light‐chain restriction, and marked endothelial injury. Clinically, GCM‐positive cases were associated with poor renal outcomes. C3G, C3 glomerulopathy; GCM, glomerular capillary microaneurysm; CL, capillary loop; CryoGN, Cryoglobulinemic glomerulonephritis; EDDs, electron‐dense deposits; En, endothelial cell; IgAN, immunoglobulin A nephropathy; IgG3, immunoglobulin G subclass 3; ITG, Immunotactoid glomerulopathy; Mo, macrophage; MC, mesangial cell; MPGN, membranoproliferative glomerulonephritis; PAL‐E, pathologische anatomie leiden endothelium; PGNMID, proliferative glomerulonephritis with monoclonal immunoglobulin G deposits; PMN, Polymorphonuclear leukocytes; PV‐1, plasmalemma vesicle‐associated protein‐1; SLE, systemic lupus erythematosus.

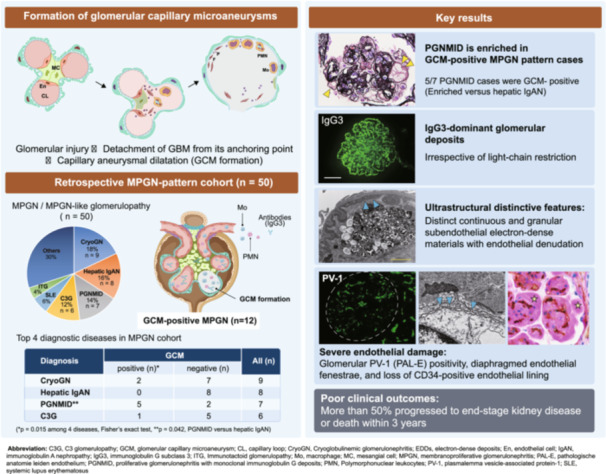

## 1 Introduction

Glomerular capillary microaneurysm (GCM) is a pathological lesion defined by aneurysmal dilatation of glomerular capillaries from the loosening or detachment of the glomerular basement membrane (GBM) from its anchoring points [1, 2]. GCM may arise from mesangiolysis, characterized by partial or complete degeneration of the mesangial cells or matrix [3]. Mesangiolysis disrupts the adhesion between the mesangium and GBM, leading to endothelial detachment, expansion of the subendothelial space. On light microscopy (LM), mesangiolysis typically appears as expanded, pale‐stained mesangial areas on periodic acid–methenamine silver (PAM) staining, which may progress to large blood cysts, ultimately resulting in GCM. Mesangiolysis may develop through several mechanisms: (i) acute mesangiolysis, often associated with glomerulonephritis (GN); (ii) endothelial injury‐driven subendothelial widening as seen in thrombotic microangiopathy (TMA); and (iii) chronic injury to the mesangial and/or endothelial cells, resulting in lamellated nodules, commonly observed in diabetic nephropathy (DN). Importantly, GCM formation is not solely attributable to mesangial cell injury but critically depends on concurrent endothelial damage.

GCM development has been demonstrated in experimental GN models, such as Thy‐1.1 GN and Habu‐snake venom–induced GN [1, 3]. In these models, mesangiolysis is initiated by mesangial cell lysis following anti‐Thy‐1.1 antibody administration or via Habu‐snake venom‐induced enzymatic degradation of the mesangial matrix. Subsequent endothelial injury disrupts the capillary architecture, promoting GCM development. In human kidney tissues, GCMs are frequently observed in nodular glomerulosclerosis in DN [4]. GCM is also observed in monoclonal immunoglobulin (Ig) deposition disease (MIDD), amyloidosis, and idiopathic nodular glomerulosclerosis [2, 3, 5]. In MIDD and AL amyloidosis, monoclonal Igs deposition in the mesangium and/or GBM disrupts their structural interface, contributing to GCM formation [6, 7]. Additionally, pathological TMA, triggered by drugs, pregnancy, or transplantation, can induce GCM via severe endothelial injury [8, 9, 10, 11]. Although GCM formation is common under these conditions, it has rarely been reported in GN. We previously reported a case of proliferative glomerulonephritis with monoclonal IgG deposits (PGNMID) presenting with a membranoproliferative GN (MPGN) pattern and multiple GCMs [12]. MPGN is a histological pattern characterized by mesangial expansion and GBM duplication on LM. Although GCMs have occasionally been observed in GN with an MPGN pattern, their clinicopathological significance has not been systematically investigated. This study aimed to elucidate the clinicopathological features of cases exhibiting an MPGN pattern with GCM.

## 2 Materials and Methods

### 2.1 Ethics

The study protocol was approved by the Human Ethics Review Committee of the Nippon Medical School (M‐2024‐221). Informed consent was obtained using the opt‐out method through the Nippon Medical School website.

### 2.2 Case Selection

**Figure 1:**
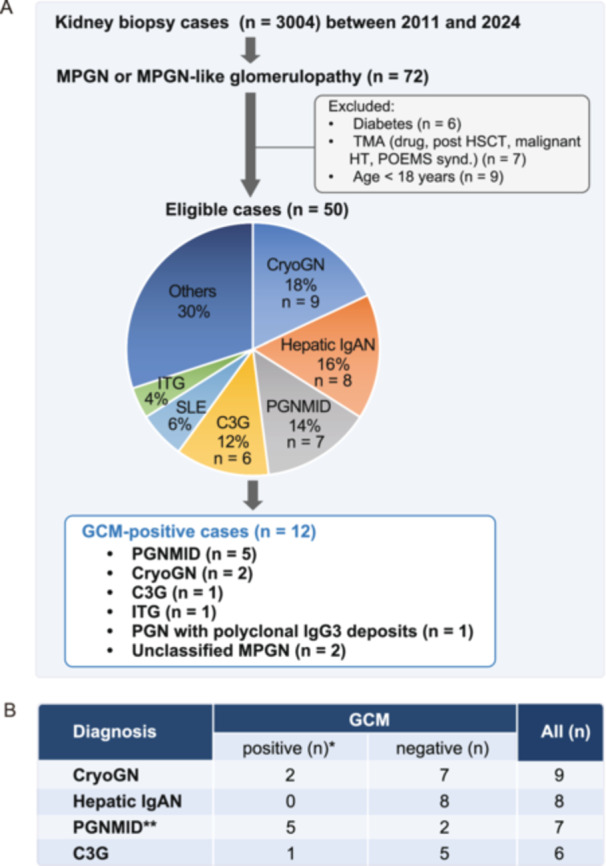
Case selection and diagnostic distribution of glomerular capillary microaneurysm (GCM)‐positive cases. (A) Among the 3004 renal biopsy cases performed between 2011 and 2024, cases diagnosed with membranoproliferative glomerulonephritis (MPGN) or MPGN‐like glomerulopathy were identified (*n* = 72). After excluding cases with diabetes mellitus (*n* = 6), thrombotic microangiopathy (TMA) (*n* = 7), and patients aged < 18 years (*n* = 9), 50 cases were considered eligible. The pie chart illustrates the diagnostic distribution of these eligible cases. Among them, 12 cases were identified as GCM‐positive, and their diagnostic breakdown is shown in the boxed panel. (B) Distribution of GCM‐positive and GCM‐negative cases across the four major diagnostic categories. GCM‐positive cases were significantly enriched in proliferative glomerulonephritis with monoclonal immunoglobulin G deposits (PGNMID) compared with other categories (*p = 0.015, Fisher's exact test with Freeman–Halton extension). Pairwise comparison demonstrated a significantly higher frequency of GCM positivity in PGNMID than in hepatic IgA nephropathy (**p = 0.042, Fisher's exact test). Abbreviations: GCM, glomerular capillary microaneurysm; CryoGN, cryoglobulinemic glomerulonephritis; PGNMID, proliferative glomerulonephritis with monoclonal immunoglobulin G deposits; C3G, C3 glomerulopathy; n, number.

Renal biopsy specimens obtained at the Department of Pathology, Nippon Medical School, between 2011 and 2024 were retrospectively reviewed. GCM was defined according to the criteria proposed by the Renal Pathology Society as “glomerular capillary dilatation due to loosening or detachment of the glomerular basement membrane from its anchoring points, typically in the context of mesangiolysis or nodular glomerulosclerosis as seen in light microscopy” [2]. Figure 1A illustrates the case selection process. Among all renal biopsy cases during the study period (*n* = 3004), cases with a pathological diagnosis of MPGN or MPGN‐like glomerulopathy were first identified (*n* = 72). Cases with diabetes (*n* = 6), pathological TMA (including drug‐related TMA, post‐hematopoietic stem cell transplantation, malignant hypertension, and Polyneuropathy, Organomegaly, Endocrinopathy, Monoclonal plasma cell disorder, Skin changes [POEMS] syndrome) (*n* = 7), and those involving patients aged < 18 years (*n* = 9), were excluded to minimize confounding factors associated with GCM formation. Diabetes mellitus is a well‐established cause of nodular glomerular lesions and GCM formation, whereas pathological TMA can cause GCM through severe endothelial injury and mesangiolysis independent of immune‐complex–mediated MPGN‐pattern injury. Patients younger than 18 years were excluded to restrict the analysis to adult‐onset MPGN‐pattern diseases, because pediatric MPGN may differ in etiology, complement abnormalities, clinical course, and treatment background. Accordingly, 50 cases were considered eligible for further analysis. Cases with GCM lesions in > 50% of the glomeruli in the biopsy specimens were classified as GCM‐positive, yielding 12 cases for the final analysis.

### 2.3 Clinical Findings and Laboratory Data

The following clinical parameters were retrospectively collected from patient medical records: age, sex, serum creatinine levels, estimated glomerular filtration rate, serum albumin levels, complement levels, immunoelectrophoresis results, urinary occult blood, urinary protein levels (expressed as g/gCr or g/day) at the time of biopsy, and past history of hypertension and/or diabetes.

### 2.4 Histopathology

Kidney biopsies were evaluated by LM, immunofluorescence (IF), and electron microscopy (EM) as routine renal pathological diagnostic procedures. For LM, tissue samples were fixed in 10% neutral‐buffered formalin, embedded in paraffin, and stained with PAM. To detect Ig and complement deposition, frozen tissue sections were processed for IF using fluorescein isothiocyanate (FITC)‐conjugated antibodies against IgG, IgM, IgA, C3, C1q, and C4 (all from MBL, Nagoya, Japan). IgG subclasses and light‐chain isotypes were also assessed using FITC‐conjugated antibodies against IgG1, IgG2, IgG3, and IgG4 (Binding Site Ltd., Birmingham, UK) and κ and λ light chains (DAKO, Carpinteria, CA). When frozen sections contained no glomeruli, IF was additionally performed on formalin‐fixed paraffin‐embedded (FFPE) sections after deparaffinization and proteolytic enzyme digestion with pronase, using the same antibody panel as that used for frozen‐section IF.

To assess glomerular endothelial cell injury, IF staining for plasmalemma vesicle‐associated protein‐1 (PV‐1/PLVAP) was performed using the Pathologische Anatomie Leiden Endothelium (PAL‐E) antibody (Progen, Heidelberg, Germany).

In addition, immunostaining for CD34 (cat. no. NU‐4A1; Nichirei Bioscience) with periodic acid–Schiff (PAS) counterstaining was performed on residual FFPE sections to evaluate endothelial lining in GCM lesions.

For EM, ultrathin sections were prepared from Epon‐embedded tissue after fixation in 2.5% glutaraldehyde and post‐fixation in 1% osmium tetroxide. Sections were stained with uranyl acetate and lead citrate, and examined under a Hitachi H‐7500 transmission electron microscope (Hitachi, Tokyo, Japan).

### 2.5 Statistical Analysis

To evaluate the distribution of GCM‐positive cases across the diagnostic categories, we first compared GCM positivity among the four most common diagnostic groups (cryoglobulinemic glomerulonephritis, hepatic IgA nephropathy [IgAN], PGNMID, and C3 glomerulopathy [C3G]). A Fisher's exact test with the Freeman–Halton extension for a 2×4 contingency table was used to assess the overall differences in GCM positivity among the groups. When the overall test was significant, pairwise Fisher's exact tests were performed with Bonferroni correction for multiple comparisons. Statistical significance was set at a two‐sided *p* < 0.05. All statistical analyses were performed using EZR (Easy R, version 1.70; Saitama Medical Center, Jichi Medical University, Saitama, Japan)—a graphical user interface for R based on the R Commander.

## 3 Results

### 3.1 GCM‐Positive Cases Across the Diagnostic Categories

After excluding cases with diabetes, pathological TMA, or patient age < 18 years, 50 cases with MPGN or MPGN‐like glomerulopathy were analyzed (Figure 1A). Although MIDD and idiopathic nodular glomerulosclerosis are well‐known conditions associated with GCM formation, no cases with these diagnoses were identified among the eligible MPGN or MPGN‐like cases. Cryoglobulinemic GN (CryoGN) was the most frequent diagnosis (*n* = 9), followed by hepatic IgAN (*n* = 8), PGNMID (*n* = 7), C3G (*n* = 6), lupus nephritis (*n* = 3), and immunotactoid glomerulopathy (ITG) (*n* = 2). The group classified as “Others” (*n* = 15) included cases with unknown etiology not falling within any of the established disease categories (*n* = 11), as well as several other distinct diagnoses represented by single cases (PV‐associated, amyloidosis, antineutrophil cytoplasmic antibody [ANCA]‐associated vasculitis, and non‐ANCA‐associated vasculitis).

Overall, 12 of the 50 MPGN or MPGN‐like cases exhibited GCM formation. Notably, GCM formation was observed in 5 of the 7 PGNMID cases, and PGNMID was the most frequent diagnosis among the GCM‐positive cases (Figure 1). Figure 1B summarizes GCM positivity in the four major diagnostic groups. Fisher's exact test (Freeman–Halton extension for a 2 × 4 table) showed a significant difference in GCM prevalence among these groups (*p* = 0.015), and after Bonferroni correction, GCM positivity was significantly higher in PGNMID than in hepatic IgAN (adjusted *p* = 0.042).

### 3.2 Pathological Findings

Among the 12 cases with GCM formation, five were classified as PGNMID, two as CryoGN, one each as C3G and ITG; the remaining three cases were unclassified MPGN‐pattern cases. Of these, Case 10 showed immune‐complex type GN with IgG3‐dominant deposits without light‐chain restriction, whereas Case 11 showed non‐IC type MPGN with unusual organized deposits and Case 12 lacked sufficient material for definitive classification (Table 1).

**Table 1:** Pathological features of GCM‐positive cases.

Case no.	1	2	3	4	5	6	7	8	9	10	11	12
Diagnosis	PGNMID with unusual organized deposits	PGNMID	PGNMID	PGNMID	PGNMID	CryoGN	CryoGN	C3G	ITG	PGN with polyclonal IgG3 deposits	MPGN with unusual organized deposits	MPGN (unknown)
IF												
IgG	(+)	(+ )	(+ )–(++)	(+ )–(++)	(++)	(−)	(+ /−)–(+ )	(+ )	(+ /−)	(+ )–(++)	(−)	(+ /−)*
IgA	(−)	(−)	(−)	(−)	(−)–(+ /−)	(−)	(−)–(+ /−)	(−)	(−)–(+ /−)	(−)	(−)	(−)*
IgM	(+/−)	(+ /−)	(+ )–(++)	(−)–(+ /−)	(+ /−)	(+ /−)–(+ )	(++)	(−)–(+ /−)	(+ )	(+ /−)	(+ /−)–(+ )	(−)*
C1q	(+)	(++)	(+ )–(++)	(+ )	(++)	(−)–(+ /−)	(+ )	(+ /−)	(−)–(+ /−)	(+ /−)	(−)–(+ /−)	(−)–(+ /−)*
C3	(+)	(+ )	(+ )	(++)	(+ )	(+ /−)〜(+ )	(+ )	(++)	(++)	(++)	(+ /−)	(−)–(+ /−)*
C4	(−)–(+ /−)	(+ /−)–(+ )	(+ /−)	(+ /−)–(+ )	(+ /−)	(−)	(+ )	(+ )	(−)	(−)	(−)	(−)*
IgG subclass	IgG3	IgG3	IgG3	IgG3	IgG3	N/A	IgG3	IgG3	N/A	IgG3	N/D	N/D
light chains	κ	κ	κ	κ	λ	κ, λ	κ, λ	κ, λ	N/A	κ, λ	N/D	(−)*
PAL‐E	Segmental (+)	(+ )	(+ )	N/A	(+ )	N/A	N/A	Segmental (+)	N/A	(+ )	segmental (+)	N/A
EM	Organized	Non‐organized	Non‐organized	Non‐organized	Non‐organized	Organized	Organized	Non‐organized	Organized	Non‐organized	Organized	Non‐organized

Abbreviations: CryoGN, cryoglobulinemic glomerulonephritis; C3G, C3 glomerulopathy; ITG, immunotactoid glomerulopathy; MPGN, membranoproliferative glomerulonephritis; N/A, not available due to insufficient tissue; N/D, not determined; PGN, proliferative glomerulonephritis; PGNMID, proliferative glomerulonephritis with monoclonal IgG deposits.

* Routine frozen‐section IF could not be evaluated because no glomeruli were present in the frozen specimen. Paraffin‐section IF was performed using antibodies against IgG, IgA, IgM, C1q, C3, C4, κ, and λ.

**Figure 2:**
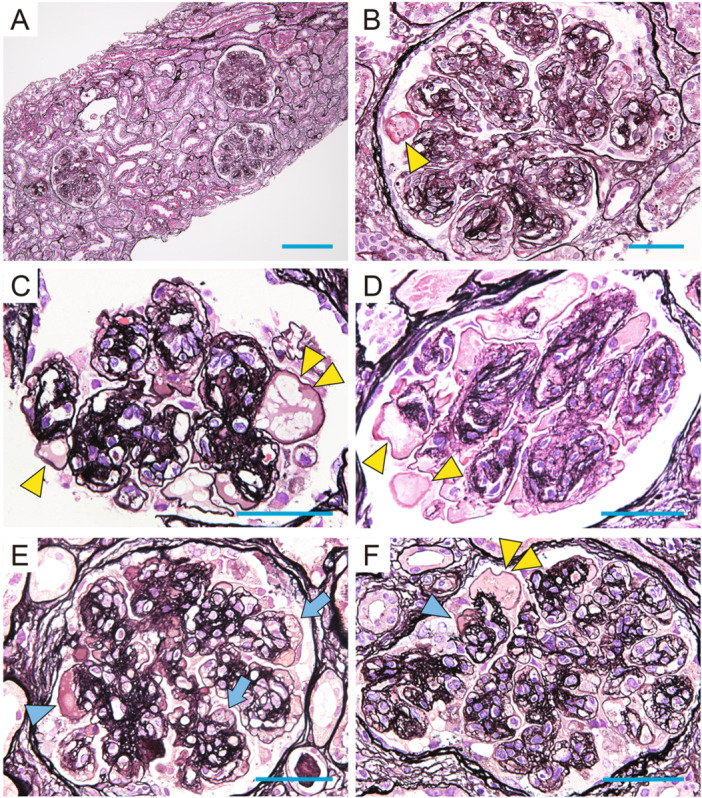
Membranoproliferative glomerulonephritis pattern with glomerular capillary microaneurysms (GCMs). Representative light microscopic findings of glomerular capillary microaneurysm (GCM)‐positive cases. Periodic acid–methenamine silver (PAM)‐stained sections from Case 3 (A, B), Case 5 (C, D), Case 10 (E), and Case 11 (F). Low‐ and high‐power views demonstrate a lobulated membranoproliferative glomerulonephritis pattern characterized by mesangial expansion, duplication of the glomerular capillary walls, and GCM formation. Yellow arrowheads indicate GCMs containing extravasated erythrocytes and plasma components without accompanying cellular elements. Light blue arrowheads indicate hyalinotic lesions. Arrows denote areas of macrophage infiltration. Scale bar: 100 µm in A, 50 µm in B–F.

LM demonstrated a predominantly lobulated MPGN pattern characterized by mesangial proliferation with duplication of the glomerular capillary walls (Figure 2A–F). Areas of endocapillary hypercellularity with inflammatory cell infiltration were also observed in some glomeruli. GCMs were frequent, presenting as markedly dilated capillary loops filled with extravasated erythrocytes and plasma proteins, with minimal or absent cellular components. Exudative lesions and foam cell infiltration were also observed. In contrast, nodular glomerular lesions typically seen in DN or MIDD were rarely identified.

**Figure 3:**
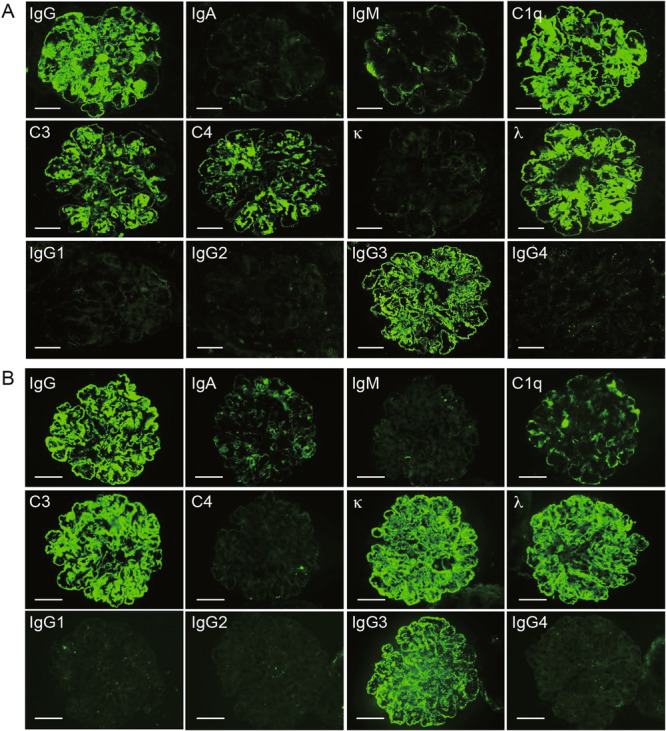
Subclass‐restricted IgG3 deposits in immunofluorescence studies. Case 5 showed intense glomerular staining for IgG, C1q, C3, and C4 (A). Subsequent subclass and light chain analysis confirmed IgG3λ restriction. Case 10 presented strong IgG and C3, characterized immune‐complex type GN. IgG subclass staining revealed monotypic IgG3 without light‐chain restriction (B). Scale bar: 50 µm.

Table 1 shows IF staining patterns for Igs and complements, and representative images from Case 5 and 10 are shown in Figure 3A and B. Among the PGNMID cases, four of five exhibited an IgG3κ pattern, whereas one showed IgG3λ deposition (Case 5 in Figure 3A). Except for Cases 11 and 12, all cases demonstrated immunological features consistent with immune complex (IC)‐type GN, characterized by concurrent positivity for Igs and complement components.

Among IgG‐positive cases with subclass staining, all showed exclusive IgG3 positivity, except for Case 9 (ITG), which was unevaluable because of insufficient tissue. Although no light chain restriction was identified in the remaining IC‐type GN cases, three cases demonstrated IgG3‐only deposition. The diagnostic categories of these three cases were heterogeneous: Case 7 was diagnosed with CryoGN, Case 8 with C3G, and Case 10 did not fit any established diagnostic category and was classified as a proliferative GN with polyclonal but subclass‐restricted (monotypic) IgG3 deposition (Figure 3B).

Case 11 showed no significant deposition of Igs or complement components. In Case 12, no glomeruli were available in the frozen specimen; therefore, routine IF evaluation could not be performed. Paraffin‐section IF was subsequently performed, however, no significant Ig or complement deposition was detected.

**Figure 4:**
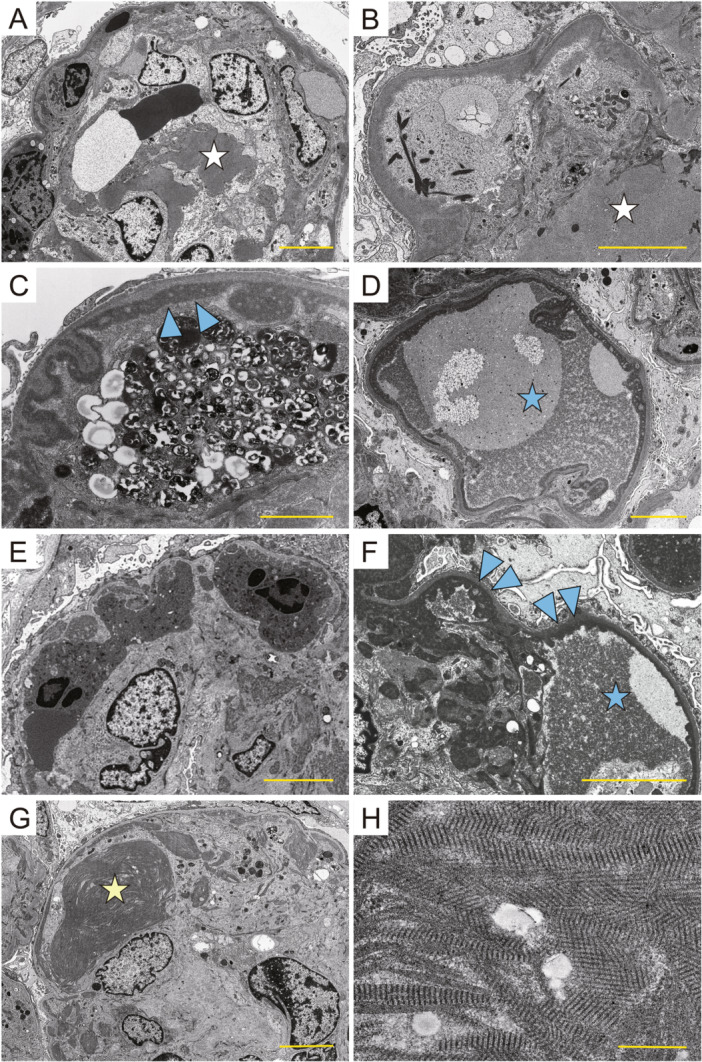
Severe endothelial cell injury with distinctive electron‐dense deposits on electron microscopy. Findings from Case 3 (A, B), Case 5 (C, D), Case 10 (E, F), and Case 11 (G, H). (A) Mesangial electron‐dense deposits (white star), macrophage infiltration and endothelial swelling with a widening of the subendothelial space. (B) Fibrin exudation within the glomerular capillary lumen and mesangial electron‐dense deposits (white star). (C) Unique, continuous fine granular subendothelial electron‐dense materials without organized structure along the glomerular basement membrane (GBM) (arrowheads), with foam cell infiltration and the loss of glomerular endothelial cells. (D) The light blue star indicates the lumen of the glomerular capillary microaneurysm (GCM) with endothelial cell denudation, in which the inner aspect of the GBM is lined by unique, continuous fine granular materials, similar to those observed in panel C. (E) Massive inflammatory cell infiltration including neutrophils into a glomerular capillary lumen. (F) The light blue star indicates the lumen of the GCM. Similar to panels C and D, unique, continuous fine granular subendothelial electron‐dense materials along the GBM (light blue arrowheads) extend from the subendothelial region into the paramesangial region. (G) The light yellow star indicates massive organized deposits predominantly located in the subendothelial region in Case 11. (H) Higher‐magnification view of the deposits in panel G, revealing a striated, organized ultrastructural pattern. The individual fibrillar or band‐like components measured approximately 16–20 nm in width and showed a periodic arrangement, with a center‐to‐center distance of approximately 40–45 nm. Scale bars: 5 μm in A, B, D–G; 2 μm in C; 500 nm in H.

The EM findings are summarized in Table 1 and illustrated in Figure 4. All cases exhibited electron‐dense deposits (EDDs), organized or non‐organized, predominantly within the mesangial and subendothelial regions (Figure 4A–H), with prominent glomerular infiltration of inflammatory cells, including macrophages, neutrophils, and foam cells. Additionally, endothelial cell injury, characterized by endothelial swelling, widening of the subendothelial space, and fibrin exudation, was observed. In several glomeruli, particularly in segments with GCM‐like changes, distinctive continuous fine granular electron‐dense materials with an irregular configuration were observed along the inner aspects of GBM with endothelial loss, some extending from the subendothelial to the paramesangial area (Figure 4C,D,F). Although these features exhibited a superficial resemblance to the finely punctate “ground‐pepper‐like” deposits characteristic of MIDD, they were morphologically distinct from each other. In Case 11, classified as non‐IC type MPGN by IF, the glomeruli demonstrated prominent striated deposits with inflammatory cell infiltration (Figure 4G,H). The individual fibrillar or band‐like substructures measured approximately 16–20 nm in width, with a periodicity of approximately 40–45 nm. Notably, in all cases, electron‐dense deposits were absent from the basement membranes of renal tubules, peritubular capillaries, and small arterioles.

**Figure 5:**
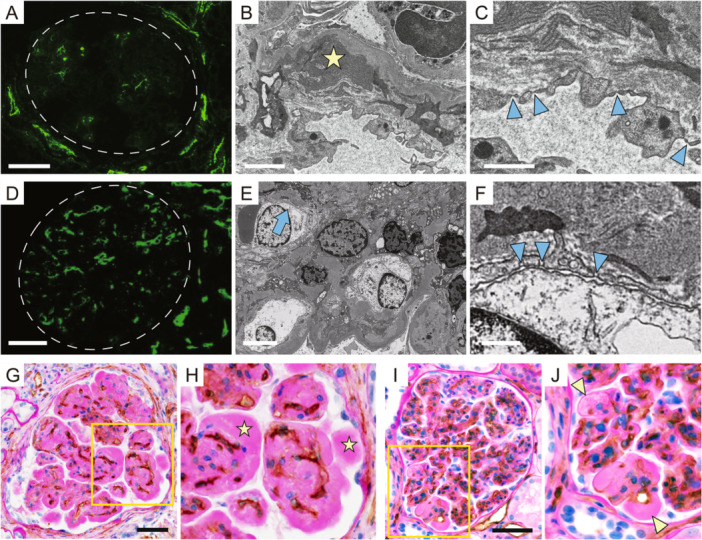
Assessment of glomerular endothelial injury in GCM‐positive MPGN‐pattern cases. PAL‐E immunostaining for PV‐1/PLVAP, electron microscopy, and CD34 immunohistochemistry were used to assess glomerular endothelial injury in GCM‐positive MPGN‐pattern cases. PAL‐E staining and ultrastructural features of fenestral diaphragms in glomerular endothelial cells are shown in Case 1 (A–C) and Case 10 (D–F). CD34 immunohistochemistry with PAS counterstaining is shown in Case 8 (G, H) and Case 9 (I, J). (A) PAL‐E staining in Case 1 showing PV‐1 expression in peritubular capillaries (PTC), with additional segmental expression within the glomerulus enclosed by the white dotted circle. (B) The light yellow star indicates unique microlamellar organized subendothelial deposits. (C) Higher magnification of panel B reveals fenestral diaphragms in glomerular endothelial cells (light blue arrowheads). (D) The area enclosed by the white dotted circle indicates a glomerulus. In Case 10, PAL‐E staining shows global positivity within the glomerulus in addition to PTC. (E) Marked inflammatory cell infiltration with endothelial cell swelling. (F) The light blue arrow in panel E indicates the area shown at higher magnification in panel F, demonstrating fenestral diaphragms in the glomerular endothelial cells (light blue arrowheads). (G, H) CD34 immunohistochemistry with PAS counterstaining in Case 8. Panel H shows a higher‐magnification view of the yellow boxed area in panel G. Stars indicate GCM lesions lacking CD34‐positive endothelial lining. (I, J) CD34 immunohistochemistry with PAS counterstaining in Case 9. Panel J shows a higher‐magnification view of the yellow boxed area in panel I. Arrow heads indicate collapsed aggregates of CD34‐positive endothelial cells within GCMs. Scale bar: 50 µm in A, D, G, and I; 2 µm in B, 1 µm in C, 5 µm in E, 500 nm in F.

Next, to assess glomerular endothelial injury, we examined PV‐1 expression and ultrastructural diaphragmed fenestrae. In Case 1, PAL‐E staining showed segmental PV‐1 expression in glomerular capillaries in addition to peritubular capillaries (Figure 5A), and EM revealed organized subendothelial deposits with diaphragmed fenestrae in glomerular endothelium (Figure 5B,C). In Case 10, PAL‐E staining demonstrated global glomerular PV‐1 positivity (Figure 5D), and EM confirmed the emergence of diaphragmed fenestrae, similar to those observed in Case 1 (Figure 5E,F). Overall, all evaluable cases exhibited global or segmental PAL‐E positivity in glomeruli (Table 1). Furthermore, CD34 immunohistochemistry with PAS counterstaining was performed in 11 of the 12 GCM‐positive cases in which residual tissue was available. Among the evaluable cases, excluding sections with extremely few glomeruli, GCM lesions consistently lacked continuous CD34‐positive endothelial lining along the PAS‐positive GBM. In some lesions, collapsed aggregates of CD34‐positive endothelial cells were observed within the GCM lesions. These findings support endothelial detachment and/or denudation as a morphological component of GCM formation. (Figure 5G–J).

### 3.3 Clinical Findings and Outcomes

The mean age was 61.6 years, with no sex predominance (Table 2). The mean serum creatinine level was 2.43 mg/dL. All patients had nephrotic‐range proteinuria, hematuria, and more than half had hypertension. One patient (Case 12) was M‐protein–positive but could not be fully evaluated and had incomplete follow‐up because of transfer, while another (Case 11) showed transient Bence–Jones proteinuria without evidence of hematologic malignancy. Serum complement levels are summarized in Table 2. Complement profiles varied across cases and were generally consistent with the underlying clinicopathologic diagnoses. Most of the patients received corticosteroids and/or immunosuppressive therapy. Within 3 years of renal biopsy, seven patients progressed to end‐stage renal disease requiring dialysis or died. Among the remaining patients, most achieved partial remission, and only one achieved complete remission. A detailed clinical course was unavailable for Case 12 due to transfer of care.

**Table 2:** Clinical characteristics of GCM‐positive cases.

Case no.	1	2	3	4	5	6	7	8	9	10	11	12	Mean value
Diagnosis	PGNMID with unusual organized deposits	PGNMID	PGNMID	PGNMID	PGNMID	CryoGN	CryoGN	C3G	ITG	PGN with polyclonal IgG3 deposits	MPGN with unusual organized deposits	MPGN (unknown)	
Age (y/o)	71	53	29	78	78	63	47	30	82	68	79	62	61.6
Sex (F/M)	F	F	M	M	M	M	F	M	F	F	F	M	6/6
sCre (mg/dL)	2.95	0.70	1.50	1.82	1.50	0.95	0.50	12.32	0.74	1.50	2.29	2.54	2.43
sAlb (g/dL)	3.3	3.1	2.0	2.9	2.3	2.3	2.7	1.4	2.3	2.6	2.6	2.7	2.5
C3 (mg/dL)	90	113	69.7	74	103	84	107	30	97	76	133	81	88.4
C4 (mg/dL)	36	26	14.8	32	30.6	1.6	10	25.7	23.2	26.9	37.3	34	24.8
M‐protein	(−)	(−)	(−)	(−)	(−)	(−)	(−)	(−)	(−)	(−)	uBJP	IgMκ	
U‐P (g/gCre)	4.9	5.1	13.2	6.9	11.0	5.6	10.9	N/A	5.9	12	9.7	7.7	8.5
U‐OB	(3 +)	(2 +)	(2 +)	(1 +)	(2 +)	(3 +)	(1 +)	(2 +)	(3 +)	(2 +)	(2 +)	(2 +)	
Hypertension	(−)	(−)	(−)	(+)	(+)	(+)	(+)	(−)	(−)	(+)	(+)	(+)	
Treatment	Steroid, MZR	Steroid	Steroid, Tac, RTX	(−)	Steroid, MZR	Steroid, PE, anti˗viral therapy	Steroid, IVCY	Steroid, MMF	Steroid, CyA	Steroid, CyA	(−)	(−)	
Prognosis	HD	ICR	CR	HD	HD, death	ICR	ICR	HD	Death	HD	HD	Unknown	

Abbreviations: CR, complete remission, CyA, cyclosporin A; CryoGN, cryoglobulinemic glomerulonephritis; C3, complement component 3; C3G, C3 glomerulopathy; C4, complement component 4; F, female; HD, hemodialysis; ICR, incomplete remission; ITG, immunotactoid glomerulopathy; IVCY, intravenous cyclophosphamide; M, male; MMF, mycophenolate mofetil; MPGN, membranoproliferative glomerulonephritis; MZR, mizoribine; PE, plasma exchange; PGN, proliferative glomerulonephritis; PGNMID, proliferative glomerulonephritis with monoclonal IgG deposits; RTX, rituximab; sAlb, serum albumin; sCre, serum creatinine; Tac, tacrolimus; uBJP, urinary Bence‐Jones Protein; U‐OB, urinary occult blood; U‐P, urinary protein.

## 4 Discussion

In this study, GCMs were identified in 24% (12/50) of cases diagnosed pathologically as MPGN or MPGN‐like glomerulopathy. PGNMID was significantly higher in the GCM‐positive group. Although GCM formation has not been emphasized as a characteristic feature in large PGNMID series, several case reports have described its occurrence [12, 13, 14, 15]. In our cohort, among the nine cases exhibiting glomerular IgG positivity, subclass analysis of eight cases showed exclusive IgG3 deposition in all. In PGNMID, IgG3κ is the most commonly observed pattern, and all five PGNMID cases with GCM in our cohort were positive for IgG3 (four IgG3κ and one IgG3λ). Furthermore, in three additional non‐PGNMID cases, IgG3 deposits alone were observed without light‐chain restriction. Therefore, GCM formation may be associated with glomerular IgG3 deposition.

IgG3 possesses unique structural and functional properties that enhance its nephritogenic potential. It exhibits the strongest C1q affinity among IgG subclasses, driving potent classical complement pathway activation [16]. Its high molecular weight and extended hinge promote glomerular deposition, while Fcγ receptor activation amplifies inflammation. Nasr et al. reported that some cases initially diagnosed as PGNMID with IgG3‐restricted deposits were subsequently shown to have both κ and λ light chain positivity [17]. These findings, consistent with our observations, suggest that the pathogenic potential of IgG3 may not depend solely on strict light‐chain clonality. Notably, IgG3‐dominant glomerular deposits can occur without detectable circulating monoclonal proteins, suggesting that oligoclonal IgG3 driven by chronic immune activation or a skewed B cell repertoire can exert pathogenic effects independent of overt monoclonal gammopathy [14, 17, 18]. In our cohort, none of the IgG3‐positive cases showed serologic evidence of monoclonality, further supporting this hypothesis. These observations underscore the importance of considering the intrinsic IgG3 pathogenicity, even in the absence of demonstrable monoclonality, and suggest that IgG subclass‐specific profiling may provide critical insights into disease mechanisms beyond traditional clonal assessment [17]. Another possible mechanism involves autoantibodies against the alternative pathway C3 convertase, including C3 nephritic factor. The production of such autoantibodies has been proposed to occur as a normal physiologic event, whereas their dysregulated or sustained production may contribute to MPGN pathogenesis; moreover, some C3 nephritic factor molecules have been reported to be of the IgG3 subclass [19]. However, these autoantibodies were not assessed in the present retrospective study. Therefore, their potential relationship with IgG3 deposition, endothelial injury, and GCM formation remains to be clarified through further accumulation of cases with detailed complement profiling.

In our cohort, PAL‐E staining revealed glomerular PV‐1 expression in all seven evaluated cases. PV‐1, a component of endothelial fenestrae and stomatal diaphragms, is normally absent from glomerular capillaries in adult kidneys and is instead confined to the peritubular capillaries and vasa recta [20, 21, 22, 23, 24]. However, in our cases, diaphragmed fenestrae were observed in injured glomerular endothelium. These findings suggest a deviation from the normal adult endothelial phenotype, potentially reflecting endothelial activation and a shift toward the remodeling phase. This interpretation is consistent with those of previous reports that PV‐1 expression and diaphragmed fenestrae reappear during fetal development or in response to endothelial injury [25, 26, 27, 28]. A recent study further demonstrated more frequent glomerular PV‐1 expression in PGNMID than in other glomerular diseases, implying more pronounced endothelial damage in PGNMID [29]. In addition, CD34 staining with PAS counterstaining further supported the presence of marked endothelial injury associated with GCM formation. In the evaluable cases, GCM lesions lacked a continuous CD34‐positive endothelial lining along the PAS‐positive GBM contours, and small collapsed aggregates of residual CD34‐positive endothelial cells were occasionally observed within the lesions. These findings suggest that endothelial detachment or denudation is an important morphological feature of GCM lesions.

In some glomeruli, unique, continuous, and finely granular subendothelial electron‐dense materials were observed along the inner capillary loops with endothelial loss. Their morphology was distinct from the powdery deposits typically seen in MIDD, which are thought to arise from interactions between monoclonal Igs, structurally abnormal light chains, and GBM components [5]. However, the precise nature of these materials cannot be determined by electron microscopy alone. As previously described in diabetic nephropathy, insudative lesions, including fibrin cap lesions, may consist of plasma‐derived proteins and lipids entrapped within glomerular capillary walls and may show fine granular or heterogeneous electron density [30]. Given that GCMs contain extravasated erythrocytes and plasma components, plasma‐derived insudative components trapped within GCMs may have contributed, at least in part, to the continuous fine granular materials observed in our cases. In this context, IgG3‐dominant deposition and associated severe endothelial injury may have promoted subendothelial insudation and retention of plasma‐derived components, resulting in the linear or continuous electron‐dense materials along the denuded inner aspect of the GBM. Further studies are required to define the molecular composition of these materials, the mechanisms underlying their formation, and their spatial relationship with IgG3 in relation to severe endothelial injury and GCM formation.

As a separate ultrastructural finding, Case 11 showed prominent striated deposits. Although their exact ultrastructural dimensions differed from those described in previous reports [31], the presence of an ordered periodic arrangement suggests that these deposits may represent organized plasma protein‐derived material. This interpretation is biologically plausible because fibrin and fibrinogen are known to form ordered arrays with characteristic banding patterns on electron microscopy [32]. Given the severe endothelial injury and GCM formation, together with IF findings showing only limited immunoglobulin and complement deposition, conventional immune‐complex–mediated glomerulonephritis is unlikely, and an organized fibrin/fibrinogen‐containing material is a plausible interpretation. This possibility is also supported by previous reports of similar unusual deposits, including a case in which mass spectrometry suggested a fibrinogen–fibronectin complex [31]. However, because the compositional data for Case 11 are not included in the present study, the precise molecular composition of these deposits is not addressed here.

The clinical course of GCM‐positive patients was characterized by poor outcomes, suggesting severe and treatment‐refractory glomerular injury. Although serologic evidence of monoclonal gammopathy was absent or inconclusive, the consistent presence of glomerular IgG3 deposits with or without light‐chain restriction, may contribute to structural and functional damage of the glomerular capillaries. However, although PGNMID was enriched among GCM‐positive MPGN‐pattern cases, the clinical significance of GCM formation within PGNMID could not be determined in the present study because the number of GCM‐negative PGNMID cases with a comparable MPGN pattern was very small. Further studies using larger PGNMID cohorts with comparable histologic patterns are required to determine whether GCM formation is associated with renal function or prognosis in PGNMID.

Beyond this limitation, several additional issues should be acknowledged. First, the single‐center retrospective design may limit the generalizability of our findings. Second, although we excluded diabetic nephropathy and overt thrombotic microangiopathy to emphasize MPGN or MPGN‐like glomerulopathies, the possibility of subtle or overlapping vascular injuries cannot be entirely ruled out. Third, IF for subclass and light chain were not feasible in all patients, and heavy/light chain IF was not available, limiting the precise classification of clonal versus polyclonal IgG3 deposition.

## 5 Conclusion

Collectively, these findings indicate that IgG3‐dominant deposition with GCM and endothelial injury defines a subset of glomerulonephritis with a more aggressive phenotype. Further studies are essential to clarify risk stratification and optimal therapeutic strategies in IgG3‐associated glomerular injury.

## Author Contributions

Akiko Mii and Akira Shimizu conceptualized the study. Akiko Mii and Akira Shimizu performed analysis and interpretation. Akiko Mii, Tomohiro Kaneko, Momoko Arai, Takehisa Yamada, Mayuko Takeuchi, Ryuji Ohashi and Yukinao Sakai were involved in data acquisition. Naomi Kuwahara, Arimi Ishikawa and Akira Shimizu were involved in methodology. Akiko Mii drafted the manuscript. Tetsuya Kashiwagi, Yukinao Sakai, Masato Iwabu and Akira Shimizu supervised the study. All authors have read and approved the manuscript.

## Ethics Statement

The study protocol was approved by the Human Ethics Review Committee of the Nippon Medical School (M‐2024‐221).

## Conflicts of Interest

Dr. Akira Shimizu and Dr. Ryuji Ohashi are Editorial Board Members of *Pathology International* and co‐authors of this article. To minimize potential bias, they were excluded from all editorial decision‐making related to the review and acceptance of this article for publication. The other authors declare no competing interests.

## Data Availability

Histopathology (HIS). Data supporting the findings of this study are available from the corresponding author upon reasonable request.
